# The NMDAR-BK channelosomes as regulators of synaptic plasticity

**DOI:** 10.1042/BST20240425

**Published:** 2025-01-28

**Authors:** Rebeca Martínez-Lazaro, Andrea Reyes-Carrión, David Bartolomé-Martín, Teresa Giraldez

**Affiliations:** 1 Departamento de Ciencias Médicas Básicas, Facultad de Ciencias de la Salud-sección Medicina, Universidad de La Laguna, Tenerife, ES-38071, Spain; 2 Instituto de Tecnologías Biomédicas, Universidad de La Laguna, Tenerife ES-38071, Spain; 3 Departamento de Bioquímica, Microbiología, Biología Celular y Genética, Facultad de Ciencias, Universidad de La Laguna, Tenerife, ES-38071, Spain

**Keywords:** functional coupling, ion channel macromolecular complexes, Large-conductance Ca^2+^- and voltage-activated K^+^channels (BK), N-methyl-D-aspartate receptors (NMDAR), synaptic plasticity

## Abstract

Large conductance voltage- and calcium-activated potassium channels (BK channels) are extensively found throughout the central nervous system and play a crucial role in various neuronal functions. These channels are activated by a combination of cell membrane depolarisation and an increase in intracellular calcium concentration, provided by calcium sources located close to BK. In 2001, Isaacson and Murphy first demonstrated the coupling of BK channels with N-methyl-D-aspartate receptors (NMDAR) in olfactory bulb neurons. Since then, additional evidence has confirmed this functional coupling in other brain regions and highlighted its significance in neuronal function and pathophysiology. In this review, we explore the current understanding of these macrocomplexes in the brain, the molecular mechanisms behind their interactions and their potential roles in neurodevelopmental disorders, paving the way for new treatment strategies.

## Introduction

Large conductance voltage- and Ca^2+^- activated K^+^ channels (KCa1.1, BK, MaxiK or slo1) are widely expressed in diverse tissues and contribute to a myriad of specialised physiological functions [1]. Specific localisation within cell compartments is a key determinant of their physiological roles [2], greatly influenced by the association with auxiliary subunits and other proteins, including other ion channels or transporters [3]. In addition to their individual roles as regulators of cellular excitability or epithelial functions, growing evidence shows that the functional association between BK and various intracellular Ca^2+^ sources underlines a general mechanism regulating Ca^2+^ signalling in very diverse physiological contexts [4–8]. Here, we review how BK channels are involved in regulating synaptic transmission by their specific association with N-methyl-D-aspartate receptors (NMDAR). The unique characteristics associated with the function and regulation of BK channels set the background for very complex physiological contexts, which may provide the molecular basis for a large diversity of physiological outputs.

### BK channels: physiological roles and significance in neuronal function

BK channels are expressed at the plasma membrane as homotetramers of α subunits encoded by the *KCNMA1* (Slo1) gene [9]. Of note, functional expression of these channels has been shown in mitochondrial and nuclear membranes, although these will not be discussed in this review [10]. BK channels play a broad range of specialised physiological roles in a variety of excitable and non-excitable tissues, including muscle, kidney, gastrointestinal tract, salivary glands and bone (recently reviewed in González-Sanabria et al. [10]). In the central nervous system (CNS), they are largely expressed in soma, axons and synaptic terminals of cells from the olfactory system, neocortex, basal ganglia, hippocampus and thalamus [11–15]. BK function in excitable tissue has been related to shaping the action potential waveforms and influencing the firing frequency in various ways. Consistent with the established understanding of potassium channel function in excitability, several studies demonstrate that blocking BK channels with paxilline (PAX), charybdotoxin or tetraethylammonium (TEA) prolongs action potential repolarisation and reduces the amplitude of the fast-duration after-hyperpolarisation (AHP) [16–20]. The pharmacological or genetic removal of BK channels can either decrease or increase the frequency of both evoked and spontaneous firing [21–25]. The latter effect can be explained by BK decreasing the latency to bring voltage-activated Na^+^ and Ca^2+^ channels out of their inactivated state, allowing higher activation frequencies [26]. Additionally, the genetic deletion of the BKβ_4_ subunit in dentate gyrus (DG) neurons led to an increase in fast AHP amplitude, sharper action potentials and higher spike frequencies [27]. These findings suggest that BK channels are not purely excitatory or inhibitory; instead, they are dynamically regulated to control neuronal excitability, depending on the cellular context [28].

**Figure 1:**
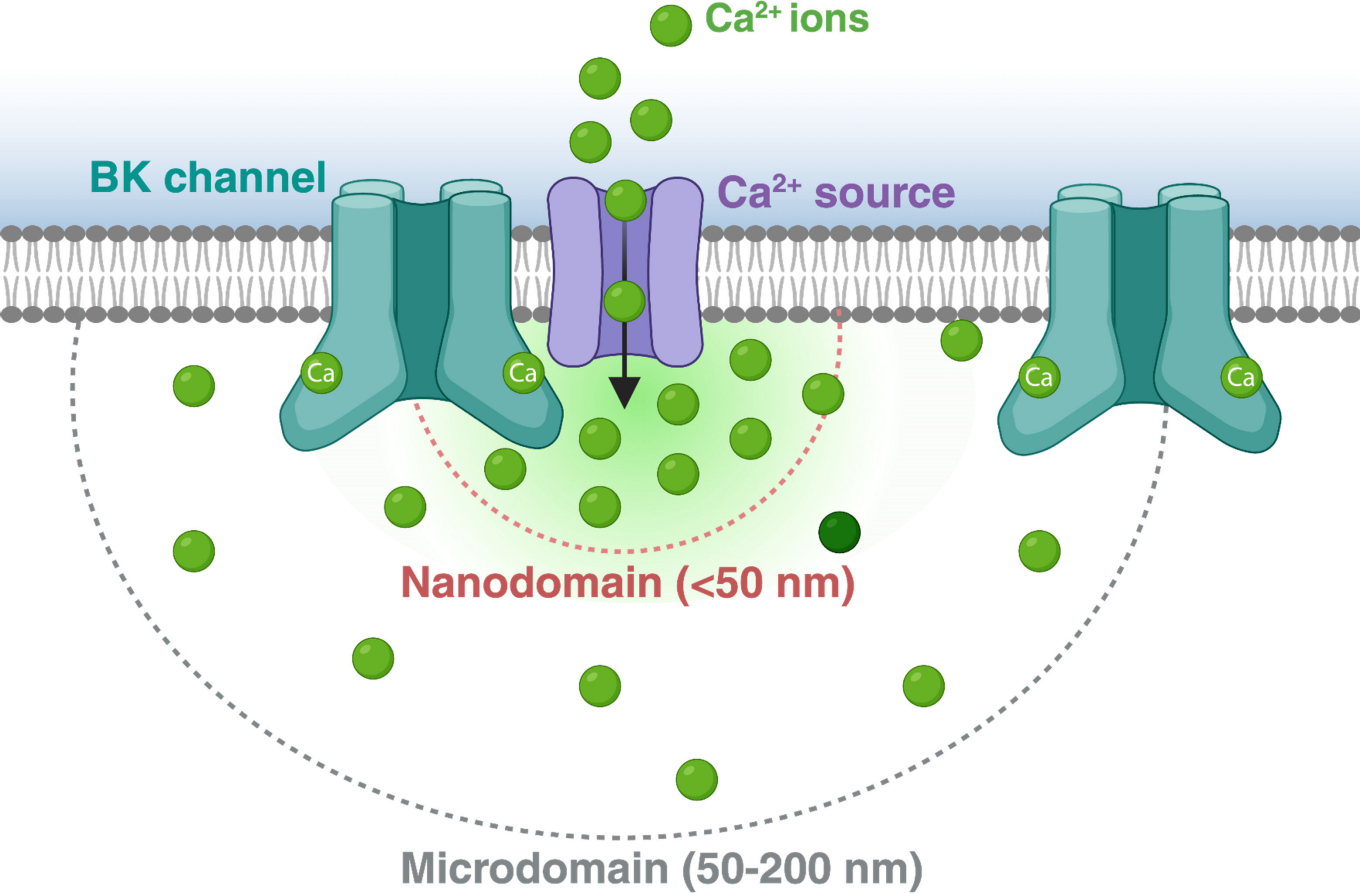
Functional nanodomains and microdomains. The biophysical features of BK channels are related with their functional association to different Ca^2+^ sources in various physiological settings. According to the distances between these different channels, such complexes (‘channelosomes’) can be defined as nanodomains (if the distance is 50 nm or lower) or microdomains (if the distance is higher than 50 nm). The association between BK and the Ca^2+^ sources is spatially and temporally regulated.

One key physiological feature of BK channel activity is the requirement of coincidental depolarisation and intracellular Ca^2+^ increase to be activated [29,30]. In fact, in many cell types, the activation of BK channels depends on the localised rise in Ca^2+^ levels reaching micromolar concentrations, which are significantly above the resting cytosolic values of 100 nm to 300 nm [31]. Thus, BK channels are commonly located in close proximity to other proteins acting as intracellular Ca^2+^ sources, associated functionally to form Ca^2+^ nano- or micro-domains (Figure 1). One such association which has been amply studied is that of BK with voltage-gated Ca^2+^ channels (VGCC). In neurons and smooth muscle cells, the synergistic membrane depolarisation from an action potential plus the Ca^2+^ entry through activated VGCC triggers nearby BK channels. The large outward K^+^ current helps repolarise the membrane, leading to the closure of the Ca^2+^ channels and ending the calcium signal [31,32]. As explained in the following sections, this negative feedback mechanism is not restricted to the functional coupling of BK to VGCC. It rather shows high versatility, including a large variety of Ca^2+^ sources [3,4]. Indeed, growing evidence shows the physiological relevance of this mechanism in a large variety of functions. These include action potential termination ([4,33] and see above), neurotransmitter release [8,34], control of circadian rhythm [35], smooth muscle contraction [36,37], cardiac sinoatrial node firing [38] and insulin secretion [39]. In line with their physiological roles, alterations in BK channels are associated with several genetically linked and acquired diseases (for recent reviews, see [6,40]).

To fully understand BK function and its connection to various calcium sources, it is important to consider the tissue-specific expression of *KCNMA1* gene products through mechanisms such as alternative splicing [1,41,42] or post-translational modifications [43]. In addition, native BK channels are commonly found in conjunction with one of two classes of modulatory subunits, β_(1-4)_ and γ_(1-4)_, which differ in structure and function [44–49]. The presence of these subunits influences nearly all aspects of BK channel gating, including its kinetics, voltage sensitivity, Ca^2+^ responsiveness and pharmacological properties [1,49].

### NMDA receptors as Ca^2+^ sources

NMDAR are heterotetrameric ligand-gated ion channels that belong to the family of glutamate-gated ion channels, also known as ionotropic glutamate receptors (iGluR), together with the α-amino-3-hydroxy-5-methyl-4-isoxazolepropionic acid receptor (AMPAR) and kainate receptor [50,51]. The function of NMDAR, which in physiological conditions mediates the inflow of Na^+^ and Ca^2+^ and outflow K^+^, has been extensively studied [52]. Since NMDARs allow the graded entry of Ca^2+^ in the cell in response to ligand binding, they play a role in synaptic plasticity, learning, memory and other higher cognitive functions [53]. The physiological relevance of NMDAR is evidenced by the fact that malfunctioning of these receptors has been related to a variety of neurological and psychiatric disorders, including Alzheimer’s disease [54], Huntington’s disease [55], stroke and schizophrenia [53], as well as major depressive disorder [56].

Seven homologous NMDAR subunits have been described so far: the obligatory GluN1/NR1 subunit, four different GluN2/NR2 subunits (GluN2A, GluN2B, GluN2C and GluN2D) and two GluN3/NR3 subunits (GluN3A and GluN3B). GluN1 and GluN3 subunits contain a binding site for the co-agonist (glycine or D-serine), whereas GluN2 subunits exhibit an agonist (glutamate) binding site. NMDAR can assemble as two GluN1 and two GluN2 subunits (GluN1/GluN2), as well as di-heteromeric (GluN1/GluN3) or tri-heteromeric (GluN1/GluN2/GluN3) combinations, resulting in the vast functional diversity of NMDAR in the CNS [52]. The expression of these genes is spatially and temporally controlled, adding to NMDAR heterogeneity throughout the brain [53].

NMDARs are highly sensitive to glutamate, with a half-maximal effective concentration in the micromolar range, and are subject to voltage-dependent blockade by Mg^2+^ ions [57,58]. In addition, their slow gating kinetics [59] and significant Ca^2+^ permeability [60,61] enable postsynaptic NMDARs to detect and interpret the coinciding activity of both pre- and postsynaptic neurons. Specifically, presynaptic release of glutamate binds to the receptor, while postsynaptic depolarisation driven by AMPARs removes the Mg^2+^ block. This simultaneous occurrence activates NMDARs, allowing Ca^2+^ to enter through the channel, triggering signalling cascades that can modulate synaptic plasticity [53]. It is then inferred that any regulatory process affecting Ca^2+^ entry through NMDAR will modify neuronal plasticity and all related effects, as we will detail in the following sections.

### The NMDAR-BK channelosome

The first evidence that Ca^2+^ entering through NMDARs could activate nearby Ca^2+^-activated potassium channels was reported in the 1980s in hippocampal neurons [62,63]. This mechanism was fully described in the olfactory bulb by Isaacson and Murphy [64], showing the activation of BK outward currents by the inflow of Ca^2+^ through glutamate-activated NMDAR. Zhang and colleagues showed that this functional association may be extended to numerous brain regions, as suggested biochemically by co-immunoprecipitation of BK and NMDAR in hippocampus, cortex, cerebellum, striatum and thalamus [65]. A role of these complexes has been reported in cortical neurons [15,66,67], hippocampal neurons [68,69], nucleus accumbens (NAc) [70], dorsal cochlear nucleus [71] and superficial dorsal horn (SDH) neurons of the thoracolumbar spinal cord [72]. Function of these associations has been tested by performing whole-cell patch-clamp recordings after applying glutamate or NMDA either towards the neuronal soma [65,67] or at dendritic locations [15,66]. Functional activation of BK by NMDAR in dendrites has been demonstrated in cortical layer 5 pyramidal neurons (L5PN) [15,66,73], as well as hippocampus CA3 and amygdala [74]. NMDAR-BK complexes have been described in both extrasynaptic and postsynaptic terminals [15,64–66,73].

**Figure 2:**
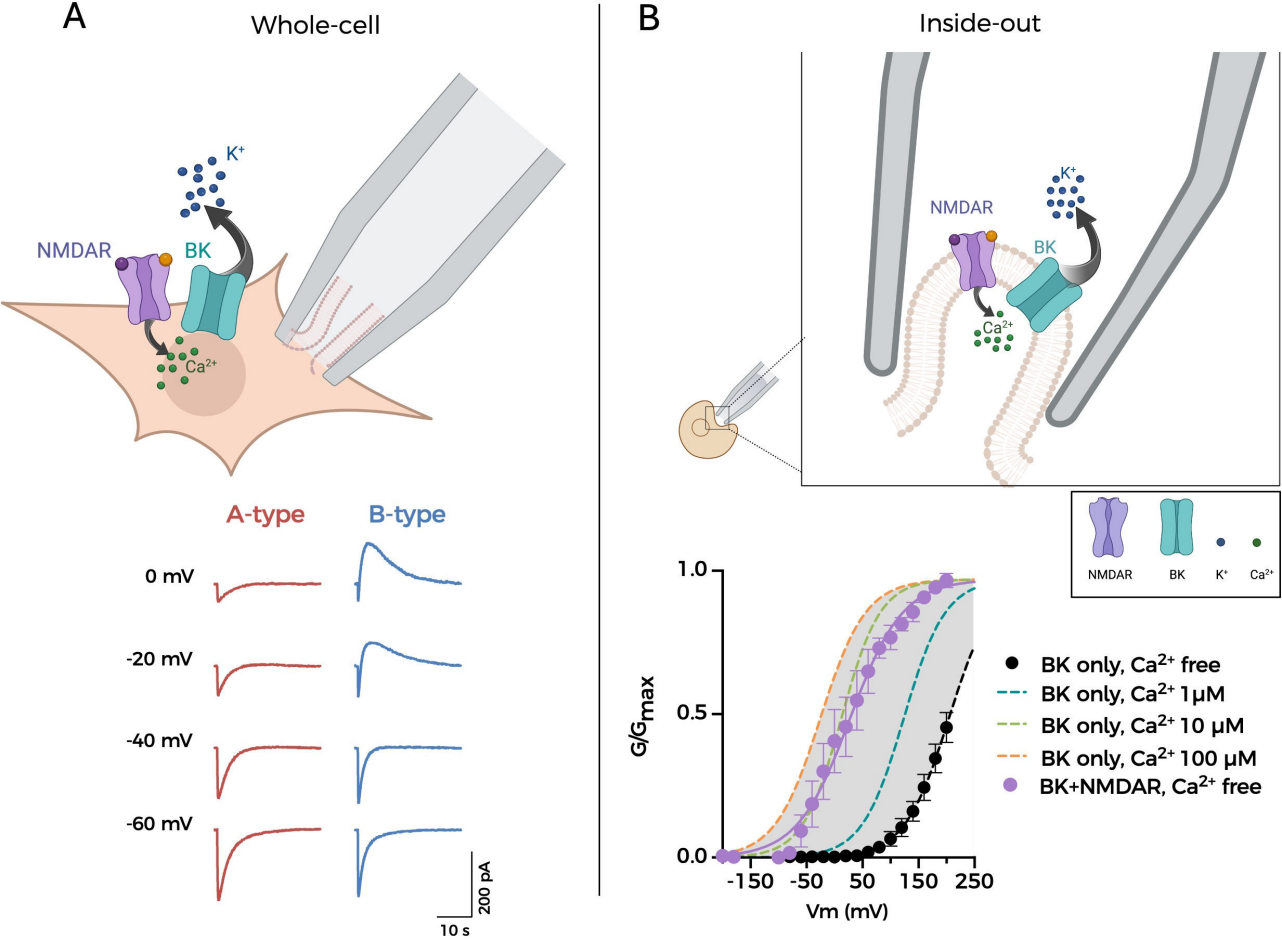
NMDAR-BK nanodomains can be reconstituted in heterologous expression systems. (**A**) Top scheme represents the cell being recorded using the whole-cell configuration of the patch-clamp technique. Bottom, representative currents recorded at different holding potentials, after the addition of NMDA or glutamate, in Mg^2+^-free solutions (see [64–66] for experimental details). ‘A-type’ cells (left, red traces) lack NMDAR-BK macrocomplexes, whereas ‘B-type’ cells (right, blue traces) express NMDAR-BK functional associations. (**B**) Top, scheme representing the inside-out excised patch configuration of the patch-clamp technique. Bottom, G-V curves obtained from patches expressing BK channels alone (black circles and coloured dashed lines) or co-expressed with NMDAR (purple circles). For experimental details, see main text and Gómez et al. [66]. Part of the data shown has been published in Gómez et al. [66].

The functional association of NMDAR and BK can be reproduced electrophysiologically in heterologous expression systems such as HEK cells, expanding the range of experimental approaches to understand the mechanisms underlying formation and function of these channelosomes [65,66]. As shown in Figure 2A, the whole-cell configuration of the patch-clamp technique can be used in transfected cells, rendering similar currents as those observed in native neurons [63–66]. Currents are recorded at different holding potentials, after the addition of NMDA or glutamate, in the absence of Mg^2+^. In those cells where NMDAR does not form functional nanodomains with BK, application of the NMDAR agonist produces inward currents (Figure 2A, ‘A-type’ recordings). In contrast, association of NMDAR to BK channels produces a slower voltage-dependent outward current at holding potentials more positive than −40 mV (Figure 2B, ‘B-type’ recordings). Further information can be inferred from the use of the inside-out excised patch configuration of the patch-clamp technique (Figure 2B). In this experimental setting, NMDARs are activated by including 200 μM NMDA and 10 μM glycine in the ‘extracellular’ pipette solution. The [Ca^2+^] in the bath can be controlled by the experimenter and is reported by the position of the BK G-V curve, which is left-shifted as [Ca^2+^]_i_ is increased [75] (Figure 2B, black circles and coloured dashed lines). Interestingly, the recordings from patches co-expressing NMDAR and BK in a bath solution containing zero [Ca^2+^] produce G-V curves that are comparable with those obtained in the presence of 10 µM intracellular Ca^2+^ (Figure 2B, purple circles). The most feasible explanation is that activated NMDARs in the patch, closely located to co-expressed BK, supply Ca^2+^ from the pipette solution. One potential caveat of this approach is that overexpressing these two proteins might lead to artifactual co-localisation. To address this, it is important to conduct appropriate control experiments with other membrane proteins or channels to assess the specificity of multicomplex formation. In support of this, we have confirmed the specificity of the NMDAR-BK interaction by showing no association between BK and Ca^2+^-permeant AMPAR variants (GluA2(Q) [76]) or between NMDAR and Kv1.1 (a voltage-gated potassium channel), under similar experimental conditions (unpublished data). In any case, and similarly to what would be done in other studies using heterologous expression systems, conclusions must take into account the limitations of this experimental model and combine it with studies in native systems. This experimental approach can be used, for instance, to evaluate the effect of different subunit combinations on the functional coupling [66]. Additionally, it constitutes a useful tool to assess the effect of mutations on BK or NMDAR affecting the functional coupling [77], as well as other remaining questions regarding the structural, biophysical and pharmacological properties of these multiprotein complexes.

### Molecular composition of the NMDAR-BK macrocomplexes

It has been proposed that the interaction between NMDAR and BK occurs via intracellular interactions between the GluN1 and BK*α* subunits [65]. Zhang et al. [65] demonstrated *in vitro* the interaction of the isolated GluN1 cytosolic regions with a synthesised peptide of the BK*α* S0–S1 loop region. In addition, these authors showed that this interaction was competitively diminished by a synthesised peptide from BK*α* S0–S1 loop [65]. These findings may suggest that, if the main interaction occurs between the obligatory GluN1 subunit and BK*α*, the role of the different GluN2 subunits would not affect the functional association. However, this aspect has not been fully addressed. Even though the proposed GluN1-BK*α* interactions may be required to form the macrocomplexes, the presence of different GluN2 regulatory subunits may introduce diversity in the biophysical properties of the nanodomains and thus in their physiological roles, such as the fine-tuning of synaptic plasticity (see below). In line with this idea, *in vivo* experiments using the excised inside-out patch technique (Figure 2B) with solutions containing physiological concentrations of Na^+^, GluN1/GluN2B NMDARs produced a larger leftward shift in the BK G-V activation curve than GluN1/GluN2A. This observation correlated with data from basal dendrites of barrel cortex L5PNs (BC-L5PNs), where the specific blockade of GluN2B-containing NMDARs resulted in a larger reduction of the NMDA-evoked outward currents [66]. Taking into account that the subunit composition of NMDARs is dynamic and varies quickly throughout development, influenced by neuronal activity or sensory experiences, even at adult synapses [53], it is tempting to speculate that differences in NMDAR composition may impact the function of NMDAR-BK complexes and therefore their effects on synaptic function.

Recordings from two distinct subpopulations of BC-L5PNs which were differentiated by the presence or absence of NMDAR-BK complexes in basal dendrites showed comparable NMDAR current characteristics, indicating a similar distribution of GluN2 subunits. Interestingly, both neuronal types showed BK channel activity. The easiest explanation for the presence or absence of NMDAR-BK complexes would be the existence of specific mechanisms targeting the channels to dendritic compartments. This could be possibly achieved by engaging specific scaffolding proteins, including the receptor for activated C kinase 1 (RACK1) and caveolin-1, both known to bind the GluN2B NMDAR subunit [78,79] and BK channels [80,81]. To address this question, protein profiling either in heterologous expression systems or native neurons would be a powerful approach to screen for candidate proteins forming the NMDAR-BK interactome [82].

Interestingly, the interaction between BK and NMDAR can be altered by GluN2B mutations related to disease. These observations show that the GluN2B mutation V618G, located in the transmembrane domain [83–85], results in weaker functional coupling, which is independent of Ca^2+^ permeation or NMDAR expression levels [77]. Strikingly, NMDAR-BK cluster quantification using superresolution microscopy shows that the size of the macrocomplexes is significantly smaller. In addition, the proportion of NMDAR and BK particles is altered in the presence of the mutation. These novel findings suggest that, even though NDMAR and BK may form complexes in different conditions, the functional characteristics of NMDAR associations are affected by the size of the clusters and by the relative number of NMDAR and BK conforming to the nanodomain [77]. It may also be inferred that the formation of NMDAR-BK macrocomplexes should not be exclusively ascribed to the proposed GluN1-BK interactions [65]. It is tempting to speculate that these alterations in the functional NMDAR-BK associations, even if partial, may be related to the pathophysiological effects of the NMDAR-GluN2B mutations. Determining the impact on NMDAR-BK function of other clinically relevant mutations in GluN2A and GluN2B subunits requires further investigation. Analogously, the effects of BK variants associated with CNS pathologies should be explored [5,40,86]. Undoubtedly, structural determination of the NMDAR-BK complexes (including the abovementioned pathological variants) would dramatically advance our understanding about the molecular interactions underlying the function and assembly of these multichannel associations.

Finally, it must be taken into consideration that the molecular mechanisms underlying NMDAR-BK interactions and function might be influenced by other factors related to the functional complexity of BK channels, including regulation by multiple auxiliary subunits (mainly *β*2 and *β*4 in the nervous system), alternative splicing variants or post-translational modifications of BK*α* [1]. The function of NMDAR-BK macrocomplexes remains to be studied in these variable physiological scenarios.

### Physiological roles of NMDAR-BK functional associations

**Figure 3:**
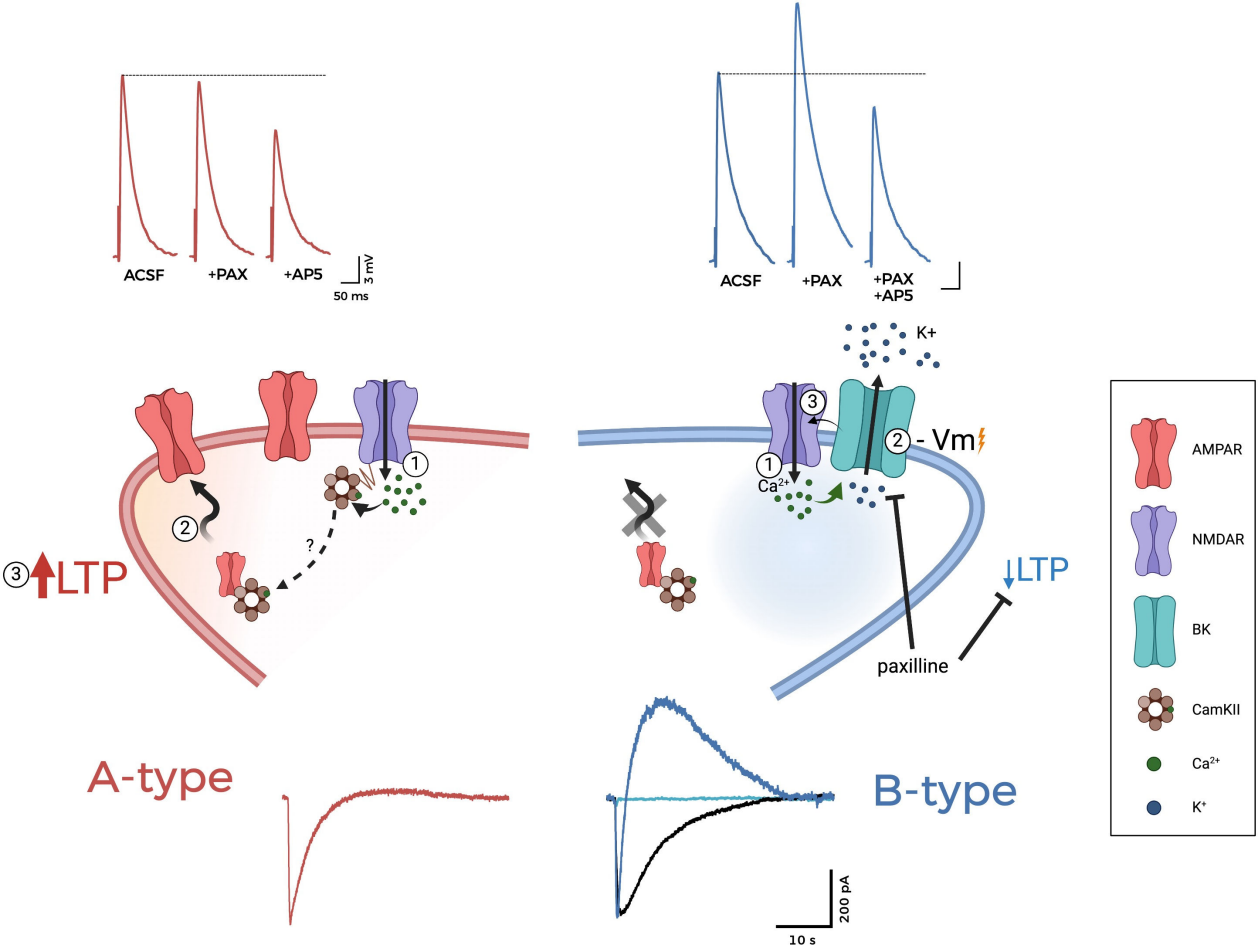
NMDAR-BK channelosomes as regulators of synaptic transmission and plasticity. Cartoon representing the biophysical and physiological features of neurons lacking dendritic NMDAR-BK multichannel complexes (‘A-type’ neurons, left, red) versus neurons containing these functional nanodomains (‘B-type’ neurons, right, blue). Inset on the right describes the molecular components shown in the cartoon. In A-type neurons (left), the entrance of Ca^2+^ through NMDAR [1] activates Ca^2+^/calmodulin-dependent protein kinase II, facilitating trafficking and stabilisation of α-amino-3-hydroxy-5-methyl-4-isoxazolepropionic acid receptors at synapses [2], therefore inducing long-term potentiation (LTP [3]). Red current traces at the bottom represent typical responses to glutamate. Red traces at the top represent synaptically evoked PSPs before and after treatment with PAX and PAX+AP5. In B-type neurons (right), Ca^2+^ entry through activated NMDARs [1] opens adjacent BK channels [2] allowing K^+^ to flow outside the cell, leading to membrane hyperpolarisation (-Vm), which restores the voltage-dependent Mg^2+^block of NMDARs [3], abolishing Ca^2+^ entry and augmenting the threshold for LTP. Blue current traces at the bottom represent typical responses to glutamate and its response to different blockers (black, PAX; light blue, PAX+AP5). Blue traces at the top represent synaptically evoked PSPs before and after treatment with PAX and PAX+AP5. Part of the data shown has been published in Gómez et al. [66]. See details in main text and Gómez et al. [66].

All the abovementioned evidence leads to the conclusion that NMDAR-BK complexes constitute a general control mechanism in the CNS, contributing to neuronal function in different brain regions [15,62–66,74]. These interactions occur both in somatic [63–65] and dendritic locations [15,66], where they may play different roles. At the soma, the NMDAR-dependent activation of BK channels provides a mechanism to regulate action potential shape and neuronal excitability, separately from dendritic input. It is worth noting that, in this subcellular context, the diverse roles of BK must be taken into account (see above), adding a layer of complexity to the proposed model. In dendrites, the coupling of NMDAR to BK may provide a negative feedback mechanism regulating synaptic transmission and plasticity (Figure 3) [15,65,66,72]. Two different physiological settings may be distinguished by the absence in dendrites of NMDAR-BK functional nanodomains (Figure 3, left, ‘A-type’ neurons) or the presence of these multichannel complexes (Figure 3, right, ‘B-type’ neurons). These neuronal types are characterised by distinct properties of their NMDA-activated currents, with important implications in their synaptic transmission and plasticity properties. In A-type neurons, the entrance of Ca^2+^ through NMDAR, which is activated by coincident glutamate binding and AMPAR-mediated depolarisation, as described above, has been proposed to activate the Ca^2+^/calmodulin-dependent protein kinase II (CamKII), which subsequently facilitates the trafficking and stabilisation of AMPARs at synapses, inducing long-term potentiation (LTP) (for extended reviews, see [53,87]). As previously described for heterologous expression systems (Figure 2), A-type neurons are characterised by NMDAR-like inward currents after addition of NMDA or glutamate to dendrites (Figure 3, bottom left panel). In these neurons, recordings of synaptically evoked post-synaptic potentials (PSPs; traces on top of the figure) showed no significant changes after blockade of BK channels with PAX (Figure 3, left, upper traces). Conversely, in B-type neurons the association of NMDAR to BK channels provides a negative feedback mechanism by which Ca^2+^ entry through activated NMDAR opens adjacent BK channels, allowing K^+^ to flow outside the cell (Figure 3, right bottom traces). The resultant membrane hyperpolarisation restores the voltage-dependent Mg^2+^ block of NMDARs, abolishing Ca^2+^ entry and augmenting the threshold for LTP [66]. A possible mechanism to explain the reduced LTP levels would be that the reduced Ca^2+^ entry results in subthreshold activation of CamKII, therefore blunting the associated increase in AMPAR trafficking. B-type neurons are characterised by NMDAR-like AP5-sensitive inward currents followed by slow outward currents (Figures 1 and 3), which are blocked by PAX (Figure 3, right; black traces at the bottom). Both inward and outward currents are abolished by AP5 (Figure 3, right; light blue traces at the bottom), demonstrating that BK activation is driven by Ca^2+^ entering through NMDARs [64–66]. Consistent with the negative feedback effect played by BK, the blockade of these channels with PAX causes an increase in the synaptically evoked post-synaptic potentials (PSPs) (Figure 3, right; upper recordings) [65,66]. A similar effect of PAX on PSPs was reported in inhibitory interneurons from lamina II of the rat spinal dorsal horn [72]. Interestingly, blocking of BK mediated by cholinergic activation in cartwheel cells of the dorsal cochlear nucleus results in enhanced excitatory post-synaptic potentials (EPSPs) and spine Ca transients [71]. In lateral amygdala (LA), reduced expression of BK channels induced by acute stress produced an increase in the evoked NMDA receptor-mediated EPSPs at the thalamo-LA synapses [88]. In BC-L5PN, it has been demonstrated that the presence of NMDAR-BK functional coupling results in reduced synaptic transmission and a higher threshold for the induction of LTP [66]. The selective plasticity attenuation exerted by NMDAR-BK macrocomplexes is restricted to the basal dendrites of these neurons since stimulation of apical dendrites did not produce any effect [15,66]. Notably, a related effect has been observed in NAc, where BK channels have been involved in LTP inhibition by ethanol (EtOH) [70]. In NAc medium spiny neurons, BK function potentiated by EtOH would more effectively counteract NMDAR activity, therefore amplifying the effect of EtOH on NMDAR [70]. Reduced synaptic transmission associated with NMDAR-BK function has been also observed in mature DG granule cells [65]. It has been proposed that this dendritic regulatory mechanism could serve to interpret the quantity and frequency of afferent synaptic inputs by selectively reducing synaptic plasticity and introducing input-specific synaptic diversity [15,66]. This process has been proposed to be further regulated by the spine structure [15].

The argument for functional interactions between NMDARs and BK channels is further bolstered by a recent study by Pham et al. [89], which revealed that BK channels facilitate NMDAR-mediated Ca^2+^ entry in hippocampal neurons, with the absence of BK activity impairing hippocampal synaptic plasticity [89]. Notably, signalling within the NMDAR-BK macrocomplexes appears to be bidirectional, as the functional components mutually influence each other. These results are relevant especially in light of findings that both gain- and loss-of-function mutations in BK have been associated with developmental delays and intellectual disabilities [90], potentially linked to disruptions in synaptic plasticity.

The influence of NMDAR-BK complexes in regulating synaptic plasticity in other brain regions such as the DG, hippocampal CA3 neurons or basolateral amygdala, where the presence of these associations has been shown [15,63–66,74], remains to be fully studied. As mentioned above, the role of NMDAR subunit composition and BK complexity (regulatory subunits, splicing variants or post-translational modifications) must be taken into account when addressing these relevant questions. For example, Ji et al. [70] found that mice lacking the *β*4 subunit showed dramatically accelerated EPSPs and reduced spike-timing dependent long-term potentiation (tLTP) [70] in the NAc, suggesting a role of these regulatory subunits in the function of NMDAR-BK complexes.

The relevance of the NMDAR-BK association has been recently demonstrated in some pathological scenarios. In the rat spinal cord, it has been proposed that BK channels interact with NMDAR to regulate visceral pain transmission and visceral hypersensitivity in a model of irritable bowel syndrome [72]. In a mouse model of Fragile X syndrome, BK-dependent synaptic integration [73] and NMDAR-BK coupling [74] are significantly altered, indicating that the integrity of this mechanism is essential in the healthy brain.

An interesting aspect that deserves further attention is the differential distribution of A-type and B-type neurons in specific brain areas. BC-L5PNs show two subpopulations with A-type (60%) or B-type (40%) features [66]. In contrast, all neurons in the CA3 hippocampus or basolateral amygdala show B-type properties [74]. The physiological implications of these neuronal population distributions on synaptic and network functions warrant further attention, as well as the exploration of other brain areas with this new functional focus.

Finally, a key question remains about the physiological roles that NMDAR-BK associations may play in different neuronal locations. In the adult brain, the majority of NMDARs located at synaptic sites are di-heteromeric GluN1-GluN2A or tri-heteromeric GluN1-GluN2A-GluN2B receptors. On the other hand, peri and extrasynaptic sites mainly contain GluN1-GluN2B receptors. In addition, GluN2C- and GluN2D-containing NMDARs can participate in synaptic transmission in some brain areas [53]. In all cases, NMDARs have been shown to be highly mobile, exchanging between synaptic and extrasynaptic sites [91]. This fact raises a very interesting question regarding the dynamics of NMDAR-BK associations. Can they form in response to neuronal activity? To this end, experiments using optogenetics could be performed to combine selected stimulation of brain areas with electrophysiological experiments discussed above. Is it possible that NMDAR-BK associations are differently shaped during development or regulated by different components or signalling pathways depending on the pathophysiological context? For instance, it has been proposed that interleukin-1*β* (IL-1*β*) may increase excitability in dissociated hippocampal neurons via regulation of NMDAR-BK function [68,69]. NMDAR-BK functional association has been suggested to be involved in the neuroprotective role of prostaglandin E2 (EP2) receptor-mediated signalling pathways in cortical neurons [67], cholinergic signalling in the dorsal cochlear nucleus [71] and inhibition of spinal opioid release [92]. Furthermore, presynaptic NMDARs have been described, which may form macrocomplexes with BK, similar to the well-studied presynaptic BK-VGCC associations [31]. Finally, the putative association of BK with non-neuronal NMDARs in astrocytes and oligodendrocytes remains largely unexplored. The role of NMDAR-BK associations in regulating synaptic transmission and plasticity seems to be attributable to their expression in postsynaptic sites, both in L5PN and dentate granular cells [15,65,66,74]. However, the expression of BK has been reported in most sites where NMDARs are expressed [53,86], suggesting that novel regulatory roles of NMDAR-BK complexes may be unveiled in the future.

### Conclusions

NMDAR-BK complexes have been known for more than 20 years, but their role(s) remain largely unexplored. Growing evidence demonstrates that these functional associations play relevant roles in the CNS, with important implications for synaptic pathologies. Further study is required to understand the molecular basis of this interaction, the location and function of these channelosomes in different CNS areas and potential roles in regulating NMDAR-dependent neuronal processes.

**Perspectives**

- N-methyl-D-aspartate receptors (NMDAR)-BK multichannel complexes constitute a regulatory mechanism of excitability and synaptic function, which is present in many neuronal types and may have been overseen in many physiological settings.
- Current roles of NMDAR-BK include the regulation of synaptic transmission and plasticity, serving as high-pass filters for incoming synaptic inputs.
- Future directions should include the characterisation of NMDAR-BK complexes in different pathophysiological settings, unveiling novel targets for neurological diseases and synaptopathies.

## Open Access

This article has been published open access under our Subscribe to Open programme, made possible through the support of our subscribing institutions, learn more here: https://portlandpress.com/pages/open_access_options_and_prices#conditional

## Abbreviations

AHP: after-hyperpolarisation
AMPAR: α-amino-3-hydroxy-5-methyl-4-isoxazolepropionic acid receptor
BC-L5PNs: barrel cortex L5PNs
BK channels: large conductance voltage- and calcium-activated potassium channels
CNS: central nervous system
CamKII: calmodulin-dependent protein kinase II
DG: dentate gyrus
EP2: prostaglandin E2
EPSPs: excitatory post-synaptic potentials
IL: interleukin
LA: lateral amygdala
L5PN: layer 5 pyramidal neurons
LTP: long-term potentiation
NAc: nucleus accumbens
NMDAR: N-methyl-D-aspartate receptors
PAX: paxilline
PSPs: post-synaptic potentials
RACK1: receptor for activated C kinase 1
SDH: superficial dorsal horn
VGCC: voltage-gated Ca2+ channels
iGluR: ionotropic glutamate receptors
tLTP: spike-timing dependent long-term potentiation
